# Low muscle mass index is associated with type 2 diabetes risk in a Latin-American population: a cross-sectional study

**DOI:** 10.3389/fnut.2024.1448834

**Published:** 2024-07-30

**Authors:** Rosario Suárez, Celina Andrade, Estefania Bautista-Valarezo, Yoredy Sarmiento-Andrade, Andri Matos, Oliver Jimenez, Martha Montalvan, Sebastián Chapela

**Affiliations:** ^1^School of Medicine, Universidad Técnica Particular del Loja, Loja, Ecuador; ^2^School of Allied Health, Eastwick College, Ramsey, NJ, United States; ^3^Escuela de Medicina, Universidad Espíritu Santo, Samborondón, Ecuador; ^4^Departamento de Bioquímica Humana, Facultad de Medicina, Universidad de Buenos Aires, Buenos Aires, Argentina; ^5^Hospital Británico de Buenos Aires, Buenos Aires, Argentina

**Keywords:** diabetes mellitus, type 2, lean body mass, low muscle mass, sarcopenia, body mass index

## Abstract

**Objective:**

Diabetes mellitus is a growing disease with severe complications. Various scores predict the risk of developing this pathology. The amount of muscle mass is associated with insulin resistance, yet there is no established evidence linking muscle mass with diabetes risk. This work aims to study that relationship.

**Research methods and procedures:**

This cross-sectional study included 1,388 employees. The FINDRISC score was used to assess type 2 diabetes risk, and bioimpedance was used for body composition analysis. Appendicular skeletal muscle mass adjusted by body mass index (ASM/BMI) was analyzed. Sociodemographic, clinical and anthropometric measures were evaluated, logistic regression models with sex stratification were conducted and ROC curves were calculated to determine the ability of ASM/BMI index to predict T2D risk.

**Results:**

It was observed that patients with higher ASM/BMI had a lower FINDRISC score in both men and women (*p* < 0.001). A logistic regression model showed and association between ASM/BMI and diabetes risk in women [OR: 0.000 (0.000–0.900), *p* = 0.048], but not in men [OR: 0.267 (0.038–1.878), *p* = 0.185]. However, when the body mass index variable was excluded from the model, an association was found between muscle mass adjusted to BMI and diabetes risk in both men [OR: 0.000 (0.000–0.016), *p* < 0.001], and women [OR:0.001 (0.000–0.034), *p* < 0.001]. Other risk factors were having a low level of physical activity, waist circumference, age and sedentary lifestyle. A ROC curve was built and the optimal ASM/BMI cut-of value for predicting T2D risk was 0.82 with a sensitivity of 53.71% and specificity of 69.3% [AUC of 0.665 (0.64–0.69; *p* < 0.0001)].

**Conclusion:**

When quantifying the risk of type 2 diabetes in both women and men, assessing muscle mass can help detect adult individuals with a high risk of developing type 2 diabetes.

## Introduction

Diabetes mellitus is a significant global health issue, with prevalence rates rising dramatically over recent decades (1). Based on data from the International Diabetes Federation (IDF), about 537 million adults had type 2 diabetes (T2D) in 2021, with projections estimating this number will rise to 643 million by 2030 and 783 million by 2045 (2). The burden of T2D is not uniformly distributed, with certain regions, including Latin America, experiencing particularly high prevalence rates. In Latin America, the prevalence of T2D is estimated to affect about 32 million people, accounting for nearly 8.4% of the adult population. This increasing trend highlights the critical need for effective prevention and early detection strategies (2).

Early diagnosis of diabetes is crucial in preventing the onset of complications and enhancing health outcomes (3). Traditional diagnostic methods, such as fasting plasma glucose and oral glucose tolerance tests, are accurate but can be resource-intensive and less accessible, especially in low-resource settings (4). Non-invasive screening tools that can be easily implemented in primary care are vital for improving early detection rates, particularly in areas with limited healthcare access. These tools are essential to ensure that more individuals are identified and managed early, thereby reducing the burden of diabetes-related complications (5).

The Finnish Diabetes Risk Score (FINDRISC) is a commonly utilized non-invasive screening tool designed to estimate the risk of developing T2D (6). FINDRISC consists of a questionnaire covering factors such as age, family history of diabetes, physical activity, waist circumference, and BMI. Research has demonstrated the applicability of FINDRISC in various populations, including those in Latin America, where it has shown promise in early detection and prevention efforts (7, 8). A recent study highlighted the effectiveness of FINDRISC in identifying individuals at high risk for T2D in Latin American and Caribbean populations, emphasizing its potential utility in these regions (9).

Skeletal muscle mass has been increasingly recognized as a crucial factor in diabetes risk. Low muscle mass is associated with insulin resistance and impaired glucose metabolism, both of which are key components in the development of T2D (10). Studies have shown that maintaining adequate muscle mass can enhance insulin sensitivity and lower the risk of diabetes (11). Given the high prevalence of diabetes and muscle mass loss in aging populations, understanding the relationship between skeletal muscle mass and diabetes risk is vital for developing effective prevention strategies (12–14).

This study aims to explore the correlation between skeletal muscle mass and diabetes risk in a Latin American population using the FINDRISC tool. By establishing this correlation, we hope to enhance prevention efforts and improve early detection of diabetes, ultimately reducing the disease burden in this high-risk population. This research offers valuable insights that can provide public health strategies and clinical practices, particularly in regions with limited healthcare resources.

## Materials and methods

### Study design and population

This is a cross-sectional analytical study. The inclusion criteria were affiliation with the Ecuadorian Institute of Social Security (IESS) and age between 18 and 75 years. Of the 1,388 employees working in various local institutions (including educational centers, hospitals, and public and private institutions), 1373 were included. Exclusion criteria were pregnancy, a diagnosis of type 2 diabetes (T2D), and cognitive impairment. The research was approved by the Ethics Committee of San Francisco General Hospital (protocol number 031). Informed consent was obtained, and participant names were replaced with unique codes to ensure anonymity.

### Sociodemographic, clinical, and anthropometric parameters

The STEPwise 3.2 method adapted to Ecuador by the Public Health Minister (MSP), National Institute of Statistics and Census (INEC), and PAHO/WHO (15), was applied in this research. Following the guidelines of this method, inquiries were made regarding sociodemographic data, tobacco and alcohol consumption. Systolic and diastolic blood pressure were measured with the participant seated using the OMRON HEM-7120 arm monitor with an accuracy of ±3% mmHg/ ± 5% pulse. Height was measured in centimeters with the patient standing erect with the head in the Frankfort plane on a portable stadiometer (Seca-217), calibrated with exact measurements in millimeters. Waist circumference was measured at the level of the navel at the end of expiration with a Cescorf tape, whose resolution is ±1 mm. BMI was calculated by bioimpedance.

### Physical activity and sedentary lifestyle

Through the IPAQ questionnaire (International Physical Activity Questionnaire), the frequency, duration and intensity of weekly physical activity were measured by MET (Metabolic Equivalent of Task), with 3.3 MET per min indicating low activity, 4 MET per min moderate activity, and 8 MET per min intense activity. The time each participant spent sitting was also recorded (16, 17).

### Risk of type 2 diabetes

The Latin American LA-FINDRISC score, as suggested in the MSP clinical practice guidelines, was applied. The risk of developing T2D was categorized as low (0–6 points), slightly elevated (7–11 points), moderate (12–14 points), high (15–20 points), or very high (more than 20 points). Following the MSP guideline recommendations, 12 points was the cut-off point to define high risk of T2D (7, 18).

### Body composition

With a multifrequency segmental analyzer that performed 10 impedance measurements (InBody 120) which has a reliability of 98%, we obtained values for weight (kg), BMI, ASM, body fat percentage, body fat mass, and visceral fat level. Additionally, we calculated the (ASM/BMI).

### Statistical analysis

Data were analyzed using IBM SPSS Statistics version 26 and EPIDAT 3.1. After assessing normality and homoscedasticity, qualitative variables were presented in frequencies and percentages, and their associations were tested using the chi-square test. Quantitative variables were expressed as means or medians and their dispersion measures. Furthermore, a one-way ANOVA followed by Bonferroni *post-hoc* tests was performed for association analysis. A multivariate analysis with logistic regression was conducted, considering the dependent variable as having or not having diabetes risk. In the unadjusted model, the independent variable considered was ASM/BMI, classifying the participants into tertiles as follows: low (0.43–0.87), moderate (0.88–1.32), and high (1.33–1.76). The model was then adjusted for other variables, including age, sedentary hours, categorical physical activity level, BMI, waist circumference, systolic blood pressure, visceral fat level, and muscle mass. Finally, a ROC curve was built to find the cutoff point of ASM/BMI that predicts T2D risk.

## Results

The characteristics of the men (*n* = 557) are summarized in Table 1. Comparative data of the variables are presented according to the ASM/BMI classified into three categories: low, moderate and high. Men with high ASM/BMI are significantly younger (32 years vs. 47 years, *p* < 0.001) and have a lower risk of T2D (6 vs. 12, *p* < 0.001). Men with high ASM/BMI have a significantly lower BMI (24.19 vs. 30.4, *p* < 0.001), smaller waist circumferences (89.4 vs. 103.5, *p* < 0.001), a significantly lower waist-hip ratio (0.90 vs. 0.97, *p* < 0.001), significantly lower systolic (121.5 vs. 128, *p* < 0.02) and diastolic blood pressure (72 vs. 79, *p* < 0.001), a lower percentage of body fat (19.2 vs. 40.2, *p* < 0.001), and a lower level of visceral fat (8 vs. 13, *p* < 0.001). Furthermore, physical activity is significantly related to moderate and high levels of ASM/BMI.

**Table 1:** Descriptive characteristics by ASM/BMI (males).

Variables		ASM/BMI			
		Low	Moderate	High	*p*-value
Age, years	Median (min–max)	47 (22–63)	39 (19–67)	32 (18–55)	**<0.001**
Smokers	n (%)	7 (5.3%)	108 (81.2%)	18 (22.8%)	0.999
Alcohol consumption, within last 30 d	n (%)	14 (4.3%)	270 (81.1%)	49 (14.7%)	0.322
Physical activity level, n (%)	Low	13 (6.4%)	174 (85.3%)	17 (8.3%)	**0.047**
	Moderate	10 (5.6%)	139 (77.7%)	30 (16.8%)	
	High	6 (3.4%)	139 (79.9%)	29 (16.7%)	
Sedentarism, hours	Median (min–max)	2 (0–10)	4 (0–16)	5 (0–14)	0.433
T2D risk (FINDRISC score)	Mean (SD)	12 (5)	9 (5)	6 (4)	**<0.001**
BMI, Kg/m^2^	Median (min–max)	30.4 (24.4–49.9)	27.4 (16.3–42.6)	24.19 (15.3–30.8)	**<0.001**
Waist circumference (WC), cm	Median (min–max)	103.5 (78.5–126)	95.2 (60.5–164)	89.4 (71–135)	**<0.001**
Systolic blood pressure, mmHg	Median (Min–max)	128 (121–160)	126 (99.170)	121.5 (84.150)	**0.018**
Diastolic blood pressure, mmHg	Median (min–max)	79 (64–96)	77 (53–114)	72 (51–97)	**<0.001**
Waist-to-hip ratio	Median (min–max)	0.97 (0.87–1.04)	0.93 (0.79–1.04)	0.90 (0.77–0.96)	**<0.001**
Body fat percentage	Median (min–max)	40.3 (23.6–52.2)	29.6 (14.2–44.8)	19.2 (9–28)	**<0.001**
Visceral fat level	Median (min-max)	13 (5–20)	12 (5–20)	8 (1–20)	**<0.001**

*P* < 0.005. ASM/BMI, apendicular skeletal muscle mass adjusted by body mass index; WC, waist circumference; BMI, body mass index.Bold values is that they are statistically significant, with a *p* value less than 0.05.

Table 2 shows the summary of women (*n* = 831) according to the proportion of ASM/BMI. In the comparative data, it is observed that women with a high ASM/BMI are significantly younger (25 years vs. 42 years, *p* < 0.001), consume less alcohol in the last 30 days (3 vs. 279, *p* < 0.001), and have significantly lower risk of T2D (4 vs. 11, *p* < 0.001). Women with high ASM/BMI have a significantly lower BMI (24.94 vs. 27.78, *p* < 0.001), smaller waist circumference (86 vs. 90, *p* < 0.001), and a significantly lower waist-to-hip ratio (0.90 vs. 0.93), a lower percentage of body fat (17.9 vs. 40.9, *p* < 0.001) and a significantly lower level of visceral fat (5 vs. 13, *p* < 0.001). Women with moderate or high ASM/BMI tend to have a greater number of hours of sedentary lifestyle, a lower level of physical activity, and slightly higher blood pressure.

**Table 2:** Descriptive characteristics by ASM/BMI (females).

Variables		ASM/BMI			*p*-value
		Low	Moderate	High	
Age, years	Median (min–max)	42 (18–75)	36 (18–75)	25 (18–75)	**<0.001**
Smokers	n (%)	45 (76.3%)	13 (22%)	1 (1.7%)	<0.075
Alcohol consumption, within last 30 d	n (%)	279 (69.9%)	117 (29.3%)	3 (0.8%)	**<0.001**
Physical activity level, n (%)	Low	351 (78.2%)	98 (21.8%)	0 (0%)	**0.042**
	Moderate	192 (77.4%)	56 (22.8%)	0 (0%)	
	High	91 (67.9%)	40 (29.9%)	3 (2.2%)	
Sedentarism, hours	Median (min–max)	4 (0–16)	6 (0–12)	5 (1–11)	**0.023**
T2D risk (FINDRISC score)	Mean (SD)	11 (0.25)	8 (0.20)	4 (3–5)	**<0.001**
BMI, Kg/m^2^	Median (min–max)	27.78 (18.6–53.2)	23.33 (17.1–32.0)	24.94 (23.9–27.64)	**<0.001**
Waist circumference (WC), cm	Median (min–max)	90 (11.2–129.5)	82.6 (62.2–108)	86 (82.2–102.5)	**<0.001**
Systolic blood pressure, mmHg	Median (min–max)	118 (88–193)	113 (80–172)	126 (110–131)	**<0.001**
Diastolic Blood pressure, mmHg	Median (min–max)	71 (45–104)	68 (50–95)	73 (70–78)	**<0.001**
Waist-to-hip ratio	Median (min–max)	0.93 (0.80–1.08)	0.88 (0.77–0.99)	0.90 (0.90–0.92)	**<0.001**
Body fat percentage	Median (min–max)	40.9 (25.8–55.3)	33.1 (16.2–44.4)	17.9 (16.9–27.7)	**<0.001**
Visceral fat level	Median (min–max)	13 (3–20)	9 (2–20)	5 (4–11)	**<0.001**

*P*< 0.005. ASM/BMI, apendicular skeletal muscle mass adjusted by body mass index; WC, waist circumference; BMI, body mass index.Bold values is that they are statistically significant, with a *p* value less than 0.05.

In women, the risk of diabetes mellitus was statistically lower by 97% when associated with the proportion of ASM/BMI [OR: 0.003 (0.001–0.010), *p* < 0.001], while in men it was statistically lower by 94% when associated with ASM/BMI [OR: 0.006 (0.001–0.002), *p* < 0.001]. After adjusting the model for age, hours of sedentary lifestyle, level of physical activity, BMI, waist circumference, systolic blood pressure, visceral fat level, and muscle mass, no association was observed between the risk of T2D and ASM/BMI in the group of men [OR: 0.267 (0.038–1.878) *p* = 0.185], while in women it remained statistically significant [OR: 0.000 (0.000–0.900), *p* = 0.048] (Table 3).

**Table 3:** Association between diabetes risk and risk factors, by sex.

	Unadjusted model		Adjusted model		Adjusted model without BMI	
	OR (CI 95%)	*P*-value	OR (CI 95%)	*P*-value	OR (CI 95%)	*P*-value
Men						
ASM/BMI	0.006 (0.001–0.002)	<0.001	0.267 (0.038–1.878)	0.185	0.000 (0.000–0.016)	**<0.001**
Age			1.058 (1.031–1.087)	<0.001	1.049 (1.023–1.076)	**<0.001**
Hours of sedentarism			1.011 (0.941–1.086)	0.770	1.002 (0.939–1.067)	0.995
Low physical activity level			1.799 (1.017–3.182)	0.044	1.795 (1.015–3.172)	**0.044**
Physical activity level, high			1.042 (0.569–1.905)	0.895	1.066 (0.584–1.946)	0.834
BMI			1.082 (1.025–2.809)	0.002		
WC			1.083 (1.026–1.141)	0.004	1.120 (1.063–1.180)	**<0.001**
Systolic blood pressure			0.998 (0.979–1.017)	0.802	1.000 (0.982–1.019)	0.966
Visceral fat level			0.973 (0.849–1.116)	0.697	0.067 (0.939–1.213)	0.320
Muscle mass			0.896 (0.523–1.535)	0.689	1.325 (1.169–1.501)	<0.001
Women						
ASM/BMI	0.003 (0.001–0.010)	<0.001	0.000 (0.000–0.900)	0.048	0.001 (0.000–0.034)	**<0.001**
Age			1.076 (1.054–1.098)	<0.001	1.076 (1.055–1.098)	**<0.001**
Hours of sedentarism			1.089 (1.028–1.153)	0.004	1.088 (1.028–1.152)	**<0.001**
Low physical activity level			2.468 (1.410–4.319)	0.002	2.490 (1.421–4.362)	**<0.001**
Physical activity level, high			1.663 (0.914–3.027)	0.096	1.688 (0.928–3.072)	0.086
BMI			1.061 (1.028–1.096)	0.459		
WC			1.061 (1.028–1.096)	<0.001	1.061 (1.028–1.096)	**<0.001**
Systolic blood pressure			1.012 (0.998–1.027)	0.096	1.012 (0.998–1.027)	0.096
Visceral fat level			0.972 (0.886–1.066)	1.066	0.975 (0.889–1.070)	0.599
Muscle mass			1.443 (0.995–2.095)	0.053	1.265 (1.117–1.433)	<0.001

*P* < 0.005. ASM/BMI, apendicular skeletal muscle mass adjusted by body mass index; WC, waist circumference; BMI, body mass index.Bold values is that they are statistically significant, with a *p* value less than 0.05.

Secondary analyses were performed in which the variable BMI was eliminated in the group of men, showing a 99% lower risk of diabetes was observed when associated with ASM/BMI in men [OR: 0.000 (0.000–0.016), *p* < 0.001]. In women, there remained a lower risk of diabetes mellitus associated with ASM/BMI [OR: 0.001 (0.000–0.034) *p* < 0.001].

Furthermore, in the adjusted model, men have a 4.9% greater risk of diabetes when associated with age [OR: 1.049 (1.023–1.076), *p* < 0.001], a 12% greater risk of diabetes mellitus when associated with waist circumference [OR: 1.120 (1.063–1.180), *p* < 0.001] and a 7.9% higher risk of diabetes mellitus when associated with less physical activity [OR: 1.795 (1.015–3.172), *p* = 0.044]. In the adjusted model, women have a 7.6% higher risk of diabetes when associated with age [OR: 1.076 (1.054–1.098), *p* < 0.001], an 8.8% higher risk of diabetes when associated with sedentary hours [OR: 1.088 (1.028–1.152), *p* < 0.001], a 2.49 higher risk of diabetes mellitus when associated with a low level of physical activity [OR: 2.490 (1.421–4.362), *p* < 0.001], and 6.1% higher risk of diabetes mellitus when associated with abdominal circumference [OR: 1.061 (1.028–1.096), *p* < 0.001].

Moreover, when the model was not stratified by sex, it was observed that there was a 99.99% lower risk of diabetes mellitus when associated with ASM/BMI in the adjusted model. When adjusted for other variables, a lower risk of diabetes mellitus persisted when associated with ASM/BMI [OR: 0.000 (0.000–0.147), *p* = 0.010] (Table 4).

**Table 4:** Association between FINDRISC score and risk factors (overall).

	Unadjusted model		Adjusted model	
	OR (CI 95%)	*P*-value	OR (CI 95%)	*P*-value
ASM/BMI	0.048 (0.026–0.002)	**<0.001**	0.000 (0.000–0.147)	**0.010**
Age			1.055 (1.009–1.102)	**<0.001**
Hours of sedentary lifestyle			1.011 (0.941–1.086)	**0.018**
Low physical activity level			2.164 (1.463–3.202)	**<0.001**
Physical activity level, high			1.364 (0.898–2.071)	0.145
BMI			0.960 (1.025–2.809)	0.650
WC			1.057 (1.031–1.085)	**<0.001**
Systolic blood pressure			0.998 (0.979–1.017)	0.802
Visceral fat level			1.021 (0.953–1.095)	0.550
Muscle mass			1.249 (1.008–1.547)	0.042

*P*< 0.005. ASM/BMI, apendicular skeletal muscle mass adjusted by body mass index; WC, waist circumference; BMI, body mass index.Bold values is that they are statistically significant, with a *p* value less than 0.05.

**Figure 1:**
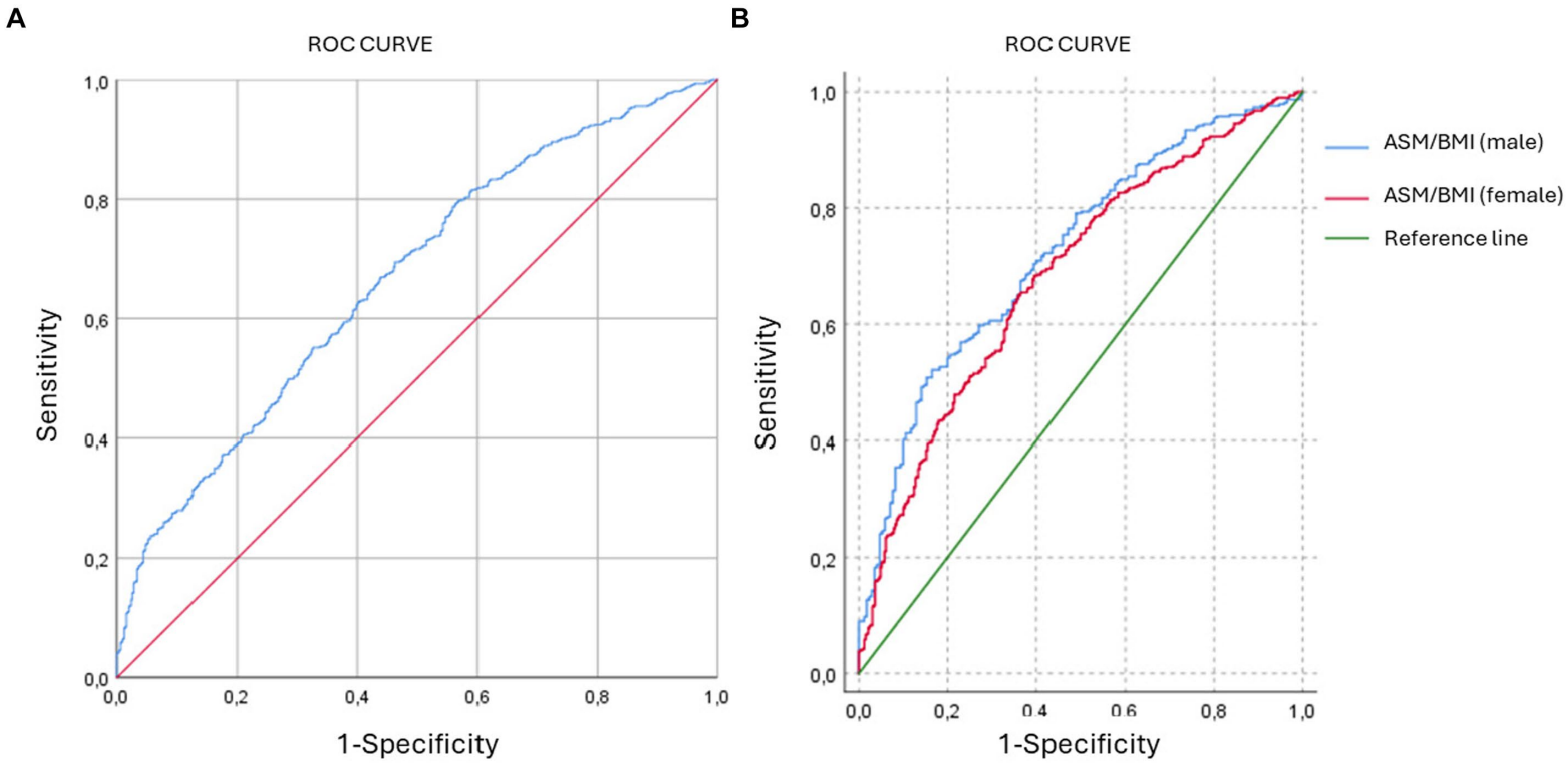
ROC curves. **(A)** ROC curve of general population. AUC = 0.665. **(B)** ROC curve of male and females. AUC = 0.723 for males and AUC = 0.684 for females.

The result of the FINDRISC score was dichotomized into those with low and slightly elevated risk versus those with moderate, high and very high risk. ROC curve was performed to determine the best ASM/BMI cut-off point that predicts having moderate or high risk in FINDRISC. Area under the curve (AUC) of 0.665 (0.64–0.69; *p* < 0.0001) was observed. With a cut-off point of 0.82 a sensitivity of 53.71% and specificity of 69.3% is obtained (Figure 1A). In men, the AUC was 0.723 (0.683–0.76; *p* < 0.0001) with a cut-off point of 1.15 with 82.94% sensitivity and 52.12% specificity. On the other hand, for women the AUC was 0.68 (0.65–0.72; *p* < 0.0001), with a cut-off point of 0.78, with 64.13% sensitivity and 64.85% specificity (Figure 1B).

## Discussion

This study evaluated the association between clinical, anthropometric, and body composition measures and the diabetes risk assessed by FINDRISC score, in 1373 participants aged 18–75 years (median 40), among whom 499 (36.4%) were at high risk of T2D. The study focused on the influence of muscle mass, as a significant part of the sarcopenia concept. Higher muscle mass, defined by the ASM/BMI index, was associated with better outcomes related to T2D risk, specifically with better BMI, waist circumference (WC), waist-to-hip ratio (WHR), systolic (SBP) and diastolic blood pressure (DBP), body fat percentage (BFP) and visceral fat level (VFL) (*p* < 0.001) in both sexes. Additionally, physical activity (PA) was significantly related to moderate and high levels of ASM/BMI in men. In women, similar results were observed, but they showed more hours of sedentary lifestyle, a lower level of physical activity, and slightly higher blood pressure.

Muscle mass is a key component in measuring sarcopenia in young adults, although muscle strength and physical performance are also important factors. Most current research has focused on studying the relationship between sarcopenia and T2D in older adults, with less reported about each sarcopenic component in younger people at risk or with risk factors for this prevalent disease (19). Recent research estimating the prevalence of obesity with low lean muscle mass (OLLMM) in adults aged 20 years and older in the US has reported an association between muscle mass and T2D and its related factors. Utilizing data from the National Health and Nutrition Examination Survey (NHANES), the study found a higher prevalence of OLLMM among Mexican-American females over 60 years old. This prevalence increases with age and is higher among individuals with prediabetes, T2D, non-alcoholic fatty liver disease (NAFLD) with fibrosis, or those who have undergone bariatric surgery (20, 21). High prevalence of sarcopenia defined using height-adjusted appendicular skeletal muscle mass (ASM/h^2^), with ASM calculated as the sum of the lean mass of the arms and legs has also been reported in the US, specifically in Louisiana, where Asians had a higher incidence of low muscle mass, compared to other ethnic groups (22).

Considering all the muscle mass indexes reported in the literature [ASM alone, ASM/height^2^ (ASM/h^2^), ASM/BMI, ASM/weight], we did not find an association between ASM/h^2^ and T2D risk, suggesting a wide divergence between the different muscle indexes. Similar results were reported in a cohort study, where absolute lower ASM was associated with incident T2D in men, but not in women (23). Another study did not find an association when analyzing both absolute ASM or ASM/h^2^ but did find one when using the ASM/BMI ratio (24). In a study including older adults with hypertension, the prevalence of low lean mass with obesity by the ASM/h^2^ index (9.8%) was lower relative to the ASM/weight (11.7%) and ASM/BMI indexes (19.6%), with the latter index evaluation being more efficient in showing muscle mass deficiency (25). Our results suggest that muscle mass index evaluation using ASM/BMI, stratified by sex, is useful to predict the risk of T2D in Latin Americans. However, there is little evidence among Latin American middle-aged adults regarding the influence of muscle and fat mass on the risk of developing T2D. Evidence does exist about a negative association between muscle mass and incident T2D (26, 27), for example, in diabetes-free Koreans, decreased skeletal muscle mass was significantly related to an incremented risk of new-onset diabetes in healthy middle-aged people. They found that the lowest sex-specific skeletal muscle mass index (SMI) tertile was significantly linked to an increased risk of developing T2D [adjusted hazard ratio (HR) = 1.31; 95% CI, 1.18–1.45] in a fully adjusted model. Presarcopenic obesity notably heightened the risk of incident diabetes (adjusted HR = 1.57; 95% CI, 1.42–1.73) compared to normal body composition, presarcopenia alone, or abdominal obesity alone. They concluded that low skeletal muscle mass, along with its coexistence with abdominal obesity, collectively incremented the risk of developing T2D, independent of glycometabolic parameters (27).

The prevalence of low muscle mass increases with age in most studies, which was also found in ours, making early detection an important issue for the prevention of T2D and many other diseases. In a recent review about sarcopenia in youth, investigators found that more than 10% of young adults in their 20s and 30s consistently had sarcopenia based on available data. Additionally, youth-onset sarcopenia seems to be more prevalent among Hispanics or Asians compared to white people and is least common in African Americans. Notably, when applying the strength criteria, more cases were identified in youth, underscoring the severity of sarcopenia in younger populations (21).

Research in Latin America related to association between T2D and body composition has been done in Chile. The authors concluded that in individuals with low muscle mass/high adiposity phenotypes showed an OR above 2 for diabetes, 2.7 for hypertension, 4.5 for metabolic syndrome, and over 2 for moderate-to-high cardiovascular risk, although the analysis included older aged participants with osteoarthritis (OAD) (28).

In our study, both males and females in the low tertile of ASM/BMI index had significantly higher T2D risk. When multivariate analyses were performed, the factor that emerged as a protective predictor for the risk of T2D was having a higher ASM/BMI. Conversely, regression models identified age, waist circumference, low physical activity level, and sedentarism as risk factors for T2D. This ASM/BMI index was also reported in a study with female participants with a history of gestational diabetes (24), and in another investigation that found that unadjusted diabetes risk was lowered by 21% in men [HR 0.79 (0.62–0.99), *p* = 0.04] and 29% in women [HR 0.71 (0.55–0.91), *p* = 0.008] for higher ASM/BMI. Nevertheless, the association ceased to be significant when age, race, smoking, education, physical activity, and waist circumference were taken into account (29).

Other authors have published a synergistic effect of low-fat mass and low muscle mass together, related to T2D, expressed in exacerbation of A1C. Interesting research among females with T2D and with overweight or obesity was reported by Terada et al., in a secondary analysis of the Look AHEAD trial, that recruited participants from 16 clinical sites across the US. This study suggests that low muscle mass has a negative effect on A1C only when combined with low-fat mass in women, which is different for men, as the latter did not show a significant effect of muscle mass on A1C and high fat mass was significantly associated with higher A1C (30).

Regarding sociodemographic factors and habits, we found that among smokers, mostly men, there was no significant association with the ASM/BMI index, even though studies reinforce the relationship of tobacco use and muscle mass loss (31). In this same bivariate analysis, alcohol consumption (in women), BMI, waist-to-hip ratio, visceral fat and fat percentage were significantly higher in people with low ASM/BMI index. Nonetheless, the association of alcohol intake with T2D risk disappeared in the regression analysis. One study reported that alcohol has no relationship with loss of muscle mass. This seems to be influenced by other factors that may intervene in the loss of muscle mass in women, such as hormonal factors (32). Furthermore, BMI has been generally reported in studies of patients with sarcopenic obesity, strengthening the relationship found in these studies, which claim that low muscle mass in populations with obesity is associated with diseases such as T2D and hypertension (33).

It is widely recognized that physical activity offers several health benefits and contributes to preventing prevalent chronic diseases while increasing muscle mass (34, 35). In this study, we identified a significant association with T2D risk, in both sexes, related to increased muscle mass. More than half of the women studied with low physical activity had low muscle mass, and in men with moderate and high physical activity levels, there was a predominance of moderate muscle mass.

In an Asian population cohort study, the authors found that predicted high lean body mass (LBM) and low fat mass (FM) were linked to a reduced risk of T2D according to anthropometric equations (36). When including patients who already have the disease, there are different analysis regarding the influence of body composition and better metabolic profiles. For example, it has been described that different combinations of fat and muscle components are associated with different outcomes, reporting that high fat and low muscle may be synergistically related to higher glycosylated hemoglobin (HbA1c) in T2D. Even with an exercise program, in participants with this profile (high fat mass, low muscle mass), exercise-induced improvements in certain cardiometabolic risk factors may be diminished (37).

In our study, higher waist circumference values are related to lower muscle mass, a result that was consistent in both sexes. Moreover, regarding body fat, we found significance in both the percentage of body fat and visceral fat in both sexes. Studies report the joint relationship of both factors with cardiovascular and other chronic diseases such as diabetes mellitus, in addition to increased mortality (38). One impact as age advances is that adipose inflammation leads to fat being redistributed toward the abdomen, infiltrating the skeletal muscles, and associated with a decrease in muscle strength, ultimately causing insulin resistance. In turn, muscle-secreted cytokines can exacerbate adipose tissue atrophy, promote chronic low-grade inflammation, and establish a vicious cycle of local hyperlipidemia, insulin resistance, and inflammation that spreads systemically, thus promoting the development of sarcopenic obesity (14, 39–41).

Additionally, the cutoff points of ASM/BMI index in identifying the risk of T2D, were higher in males than in females, with higher sensitivity in men but more specificity in women. Some previous research has developed ROC curve analysis to determine cut-off points of various anthropometric variables to predict metabolic diseases, such as type 2 diabetes mellitus, gestational diabetes or metabolic syndrome (42–44), and also using indices such as the fat-muscle ratio (FMR), among others. One of the studies that evaluated this FMR in women tried to predict the risk of gestational diabetes with a cut-off value of 1.305 (45). Another study described different anthropometric indices such as Body Roundness Index (BRI), body shape index (ABSI), and lipid accumulation product to predict metabolic syndrome among industrial workers in Russia (46). However, no cut-off points have been reported for the ASM/BMI for either sexes related to diabetes risk, as in our study, so it could be suggested as a possible anthropometric marker to predict this risk.

Finally, the ASM/BMI may serve as a convenient parameter for screening individuals at high risk for T2D, especially among males.

This study has several limitations. First, although we utilized BIA to evaluate body composition, which is not the gold standard method, it has been validated as a non-invasive method that offers precise estimates of skeletal muscle mass, which closely align with measurements obtained through DXA and magnetic resonance imaging across different ages, volume statuses, and BMI ranges (47, 48). Second, we could not assess the role of muscle strength or quality and laboratory variables such as glycemia and lipid profile to T2D risk. Additional research is needed to elucidate the connection between muscle strength, laboratory parameters, and metabolic risk in young Latin American adults. Third, because this study is observational in nature, the cross-over design does not favor causal relationships. Therefore, prospective studies would be needed for internal and external validation of ASM/BMI index and to be able to use it routinely, in that case, it would be a proposal that could contribute to the early detection of people at risk of diabetes mellitus based on novel indicators.

## Conclusion

In summary, muscle mass determined by the ASM/BMI index was associated with the risk of type 2 diabetes in middle-aged Ecuadorian men and women, and exercise appeared to be the best parameter to reduce this risk. The strongest factor associated with this risk was having a low level of physical activity, followed by waist circumference, age and sedentarism.

When quantifying the risk of type 2 diabetes in women and men, doctors may find that assessing muscle mass will help detect adults at an incremented risk of developing type 2 diabetes. Aerobic and resistance exercise can contribute to preventing diabetes by increasing muscle mass, which should be further investigated in interventional studies.

## Data availability statement

The raw data supporting the conclusions of this article will be made available by the authors, without undue reservation.

## Ethics statement

The studies involving humans were approved by the Human Research Ethics Committee of the San Francisco Hospital in Quito. The studies were conducted in accordance with the local legislation and institutional requirements. The participants provided their written informed consent to participate in this study.

## Author contributions

RS: Conceptualization, Formal analysis, Writing – original draft, Writing – review & editing, Data curation, Supervision, Validation. CA: Conceptualization, Formal analysis, Writing – review & editing, Validation. EB-V: Conceptualization, Data curation, Formal analysis, Validation, Writing – review & editing. YS-A: Conceptualization, Methodology, Validation, Writing – review & editing. AM: Formal analysis, Validation, Writing – review & editing. OJ: Methodology, Validation, Writing – review & editing. MM: Supervision, Validation, Writing – review & editing. SC: Formal analysis, Supervision, Validation, Writing – review & editing.

## Glossary

ANOVA: Analysis of variance
ASM: Appendicular skeletal muscle mass
ASM/h^2^: Height-adjusted appendicular skeletal muscle mass
AUC: Area under the curve
BFP: Body fat percentage
BIA: Bioimpedance analysis
BMI: Body mass index
BP: Blood pressure
DBP: Diastolic blood pressure
DXA: Bone densitometry
FINDRISC: Finnish Diabetes Risk Score
FM: Fat mass
HbA1c: Glycosylated hemoglobin
IBM: International Business Machine
IESS: Ecuadorian Social Security Institute
INEC: National Institute of Statistics and Census
IPAQ: International Physical Activity Questionnaire
LBM: Lean body mass
MET: Metabolic Equivalent of Task
MSP: Public Health Minister
NAFLD: Non-alcoholic fatty liver disease
NHANES: National Health and Nutrition Examination Survey
OAD: Osteoarthritis
OLLMM: Obesity with low lean muscle mass
OR: Odds ratio
PA: Physical activity
SBP: Systolic blood pressure
SPSS: Statistical package for social sciences
T2D: Type 2 diabetes
US: United States
VFL: Visceral fat level
WC: Waist circumference
WHO: World Health Organization
WHR: Waist-to-hip ratio
